# Formulation and Characterization of an Effervescent Hydrogen-Generating Tablet

**DOI:** 10.3390/ph14121327

**Published:** 2021-12-18

**Authors:** Moritz Rosch, Kurt Lucas, Jozef Al-Gousous, Ulrich Pöschl, Peter Langguth

**Affiliations:** 1 Multiphase Chemistry Department, Max Planck Institute for Chemistry, 55128 Mainz, Germany; k.lucas@mpic.de (K.L.); U.poschl@mpic.de (U.P.); 2 Department of Biopharmaceutics and Pharmaceutical Technology, Institute of Pharmaceutical and Biomedical Sciences, Johannes Gutenberg University Mainz, 55128 Mainz, Germany; joalgous@uni-mainz.de; 3 Department of Pharmaceutical Sciences, University of Michigan, 428 Church Street, Ann Arbor, MI 48109, USA

**Keywords:** tablet, hydrogen, effervescent, roller compaction, dynamic vapor sorption, stability, release, saccharide, filler, antioxidant

## Abstract

Hydrogen, as a medical gas, is a promising emerging treatment for many diseases related to inflammation and oxidative stress. Molecular hydrogen can be generated through hydrogen ion reduction by a metal, and magnesium-containing effervescent tablets constitute an attractive formulation strategy for oral delivery. In this regard, saccharide-based excipients represent an important class of potential fillers with high water solubility and sweet taste. In this study, we investigated the effect of different saccharides on the morphological and mechanical properties and the disintegration of hydrogen-generating effervescent tablets prepared by dry granulation. Mannitol was found to be superior to other investigated saccharides and promoted far more rapid hydrogen generation combined with acceptable mechanical properties. In further product optimization involving investigation of lubricant effects, adipic acid was selected for the optimized tablet, due to regulatory considerations.

## 1. Introduction

In recent years, molecular hydrogen has found increasing use as a medical gas that exerts benefits in many diseases and under diverse physiological conditions [1,2,3,4]. Since a groundbreaking publication by Ohsawa and colleagues in 2007 [5], several hundred peer-reviewed papers have proven the pharmacological effects of molecular hydrogen [3,6]. Initially, positive effects were mainly demonstrated in cell culture experiments and animal studies, e.g., in models of Parkinson’s disease [7,8]. Subsequently, several clinical studies confirming the positive effects of hydrogen gas in acute cerebral infarction [9], ischemic stroke [10], hemodialysis [11], type 2 diabetes [12], and metabolic syndrome [13] have been published. Moreover, molecular hydrogen can enhance ergogenic effects in athletes [14,15,16]. We recently proposed that molecular hydrogen may also be beneficial for the treatment of acute and chronic fatigue [17]. The spectrum of diseases for which hydrogen has been shown to be effective is broad, due to the fact that many diseases are associated with inflammation and an enhanced production of reactive oxygen and nitrogen species (ROS/RNS) [18,19,20]. The beneficial effects of molecular hydrogen involve activation of the cell’s antioxidant system via the transcription factor Nrf2 [6]. In particular, it has been shown to reduce oxidative stress in mitochondria [21]. Besides its antioxidative attributes, anti-inflammatory effects [1,22,23] have also been attributed to hydrogen.

Molecular hydrogen is administered via inhalation, injection of hydrogen-enriched saline, or drinking of hydrogen-enriched water [24], with the latter being the most convenient form for human consumption. Hydrogen-enriched water can be generated by solving and dispersing the gas under pressure, electrolysis, or by an effervescent hydrogen-generating tablet. Our aim was to develop and characterize an effervescent tablet that generates hydrogen and is of sufficient hardness to allow handling during further processing. Furthermore, our goal was to develop a tablet that meets European regulatory requirements for nutritional supplements. Tablets have many advantages compared with other preparation methods, such as flow-through of gas or electrolysis, and can also be prepared by non-professionals as a way to administer hydrogen-enriched water safely and freshly. Moreover, tablets would require substantially less transport and storage capacity compared to what is required for hydrogen-enriched water. Furthermore, storage of hydrogen-enriched water requires special, very dense metal bags which are harmful for the environment. The developed tablets also make hydrogen accessible for a broad range of users who cannot afford the expensive instrumentation required to prepare hydrogen-enriched water. Finally, the tablets can be used independently of an electric power source.

Regular pharmaceutical effervescent tablets contain effervescent agents that react upon contact with water and generate carbon dioxide, allowing for a rapid release of the active pharmaceutical ingredient [25,26,27,28,29]. Moreover, oxygen-releasing, anti-microbial effervescent tablets have already been described in the literature [30]. Since this hydrogen-generating tablet represents a new kind of effervescent tablet, we were confronted with several challenges during development. Compaction of effervescent tablets is generally considered as technically challenging [31], because the effervescent components as such constitute an incompatible mixture within the tablet. Effervescent components are usually hygroscopic and moisture-labile since water uptake can trigger effervescent reactions during processing and storage. Furthermore, water uptake of the granules before and during tableting can result in the tablets sticking to the punches of the tableting machine. Therefore, one aspect of this work focused on the careful selection of excipients and processing conditions to increase the formulation’s resistance and minimize the formulation’s exposure to humidity. Further challenges such as segregation of the excipients [32,33] during the first attempts at direct compaction, sticking during compaction [34,35], slow disintegration, as well as regulatory restrictions regarding—for example, excipient selection—also had to be overcome. Moreover, the determination of hydrogen availability from an effervescent tablet required establishment of a novel non-pharmacopeial method. This method is well suited for the quality control of effervescent hydrogen-generating tablets while being potentially applicable to other effervescent tablets as well. In addition, this study provides insights regarding the use of saccharide-based fillers in effervescent tablets manufactured using roller compaction.

## 2. Results

To develop a hydrogen-generating effervescent tablet based on the chemical redox reaction of an acid and metal, which was the aim of this work, a substance which can reduce the acid’s protons by virtue of its lower reduction potential compared to hydrogen is required. Furthermore, both components need to exhibit the exact degree of chemical reactivity which is needed in order to ensure a safe chemical reaction upon contact with water and to ensure sufficiently rapid tablet disintegration within a few minutes. Of course, both components must also be well tolerated physiologically. Suitable candidates include alkali metals such as sodium, alkaline earth metals such as calcium, or even transition metals such as iron. For these reasons a solid acid was chosen as a proton donor, which is commonly found in effervescent tablets [36]. Specifically, elementary magnesium was selected, as it reduces protons to hydrogen gas in an acidic medium. Metals such as sodium were not considered to be safe because of their extremely high chemical reactivity, whereas iron was regarded as chemically too inert. Regarding organic acids, the best results were obtained with ascorbic acid and Citrocoat^®^ N.

### 2.1. Selection of Tableting Excipients

In contrast to a conventional tablet, the formulations described here generate hydrogen instead of carbon dioxide from a chemical redox reaction [37,38,39,40,41,42,43] in which elementary magnesium reduces hydrogen in an acidic medium according to the equation

(1) $$\overset{0}{\mathrm{Mg}}+2\;\overset{+I\;-I\;}{\mathrm{HAc}}\;\Rightarrow \overset{+\mathrm{II}\;-I\;}{{\;\mathrm{MgAc}}_{2}\;}+\overset{0}{{\;H}_{2}}$$

Magnesium powder of very high purity and small particle size was selected to ensure a quick chemical reaction. With Citrocoat^®^ N (granule with citric acid core coated with a monosodium citrate shell), a functional acid was selected that enables a rapid effervescent reaction that pure citric acid (pk_a_1 = 3.14, pk_a_2 = 4.77) would provide and offers a reasonable hygroscopicity by dint of its less hygroscopic monosodium citrate shell. Additionally, effervescent tablets need to disintegrate quickly upon contact with water and since they form oral solutions, taste is an important consideration during product development. Therefore, saccharides and saccharide-derived fillers are often used in such formulations. In addition to their high water solubility and acceptable taste, these excipients tend to exhibit favorable compaction properties. Therefore, one of the main objectives of this study was to evaluate different saccharide fillers and determine their impact on tablet properties. Highly purified ascorbic acid contributes to the physiological antioxidant effects of the tablet and can also protect the magnesium powder in the formulation from oxidation during processing and storage. The water-soluble lubricants, sodium stearyl fumarate and processed adipic acid, were chosen to avoid delayed disintegration, which would especially occur in acidic media [44], as well as fatty acid layers on top of the effervescent solution created by water-insoluble lubricants like magnesium stearate. 

### 2.2. Disintegration, Porosity, Kinetic Hydrogen Generation, and Magnesium Content

**Figure 1:**
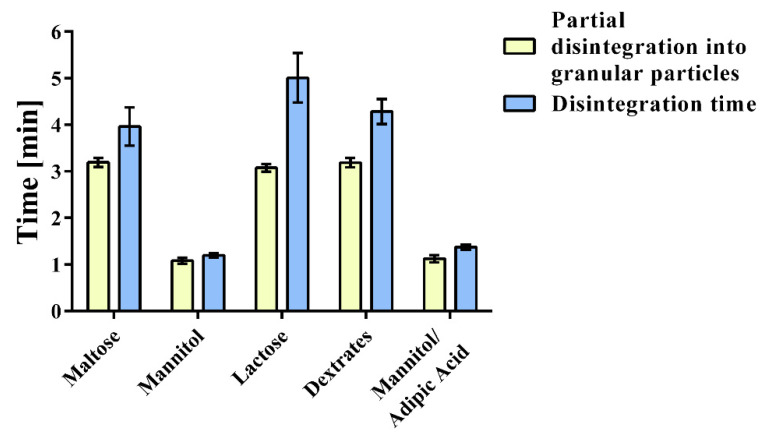
Disintegration time of tablets containing different fillers. Experiments were conducted in a beaker with 200 mL of water as described in the monograph of effervescent tablets in the Ph. Eur. 10.1/0478. Primary and secondary endpoints of disintegration were determined visually. The first measurement was taken when the tablet had partially disintegrated into granular particles (DiG = partial disintegration into granular particles). The second measurement was taken as the endpoint, after the tablet and the particles had disintegrated completely (DT = disintegration).

The disintegration of the mannitol-based tablets was significantly faster than with other fillers (Figure 1, Table 1) with an average disintegration time of 72 ± 3 s (mannitol) and 83 ± 3 s (mannitol/adipic acid), respectively. Interestingly, all fillers bar mannitol took a longer time to disintegrate completely, after partially disintegrating into granular particles. The disintegration into the granular particles took roughly 3 min for maltose, lactose, and dextrates, while the complete disintegration time was 238 ± 25 s for maltose and 257 ± 15 s for dextrates. It took 300 ± 32 s for the lactose-based particles to disintegrate completely, which is significantly longer than for every other formulation. Thus, 116 s was required for the disintegration of the granular lactose particles, while this step was significantly shorter for the maltose-based (46 s) and dextrates-based (66 s) particles and only 15 s or less was required for the complete disintegration of the mannitol-based formulations (7 s for mannitol and 15 s mannitol/adipic acid, respectively).

In conjunction with fast tablet disintegration times, hydrogen generation was also relatively fast for the mannitol-based tablets. They generated more than 80% of the theoretically possible hydrogen content after only 100 s. At this time, the tablets with dextrates as a filler had generated slightly more than 7% of the theoretically possible hydrogen amount.

**Figure 2:**
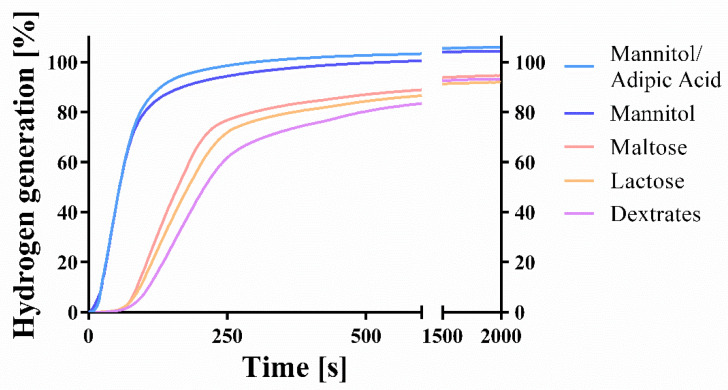
Kinetic hydrogen generation measurement (means; *n* = 3). Kinetics of hydrogen generation were measured using different fillers and lubricants. Means were calculated and plotted against time. SD values can be found in Table 1.

The amount of generated hydrogen ranged from 91.93 ± 1.59% (lactose) to 105.91 ± 2.08% (mannitol/adipic acid) (Figure 2; Table 1) of the theoretically possible amount assuming a complete chemical reaction. The mannitol-based formulations generated significantly more hydrogen than the other fillers. No significant differences were observed between mannitol-based tablets. After adjustment for differences in tablet weight, total hydrogen generation averaged 102.8 ± 2.93% (mannitol) and 103.89 ± 1.77% (mannitol/adipic acid), respectively. Moreover, the magnesium content was determined by complexometric titration of magnesium ions (Table 1). The magnesium content ranged from 100.54 ± 1.70% (lactose) to 106.88 ± 1.95% (dextrates). Only the values of the mannitol-based batches (104.03% and 104.94%) suggest a complete oxidation of the magnesium, leading to hydrogen generation.

The f2 comparison suggests that hydrogen generation curves with a dimensionless value of above 50 can be considered as similar according to SUPAC guidelines (Table 2). The following curves are accordingly considered to be similar: mannitol and mannitol/adipic acid (f2 = 85.95); maltose and lactose (f2 = 64.24); lactose and dextrates (f2 = 54.65).

**Figure 3:**
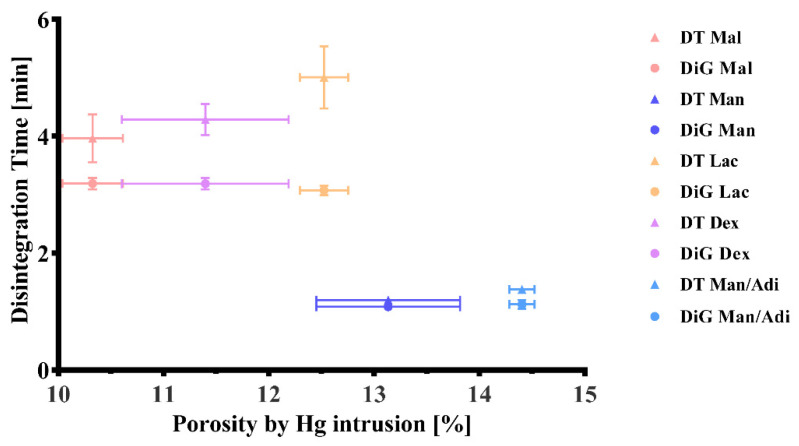
Disintegration time vs. porosity. Experiments were conducted in a beaker with 200 mL of water as described in the monograph of effervescent tablets in the Ph. Eur. 10.1/0478. Primary and secondary endpoints of disintegration were determined visually. The first measurement was taken when the tablet had partially disintegrated into granular particles (DiG = partial disintegration into granular particles). The second measurement was taken as the endpoint, after the tablet and the particles had disintegrated completely (DT = disintegration). These values were recorded for different fillers (Mal = maltose; Man = mannitol; Lac = lactose; Dex = dextrates; Man/Adi = mannitol/adipic acid) and were plotted against the porosity of the tablets, which was measured by mercury intrusion.

**Figure 4:**
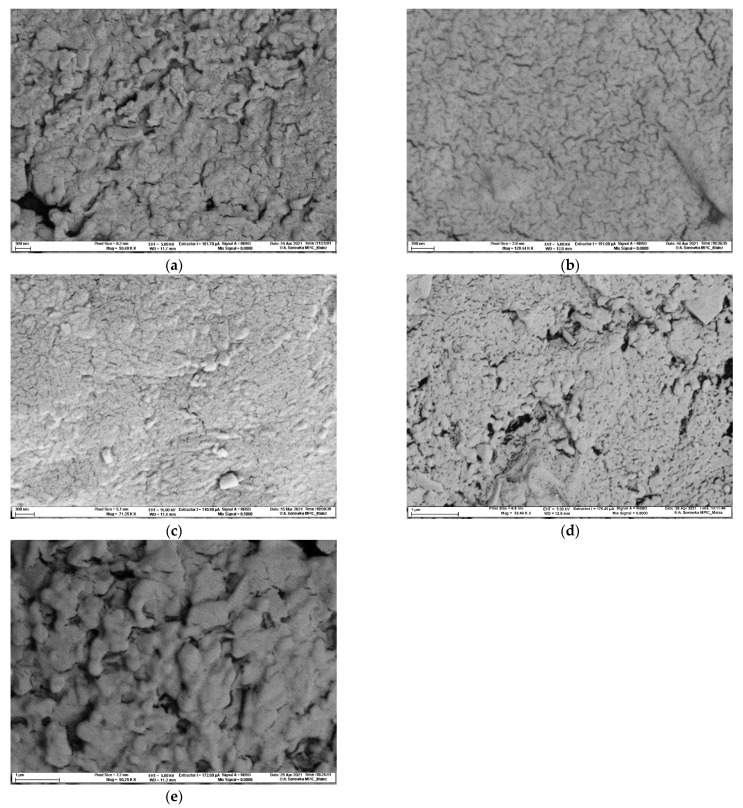
Scanning electron microscope pictures of (**a**) the maltose-based batch (compaction force 40 kN; magnification: 58,480×); (**b**) the mannitol-based batch (compaction force 40 kN; magnification: 129,440×); (**c**) the mannitol/adipic acid-based batch (compaction force 25 kN; magnification: 71,350×); (**d**) the lactose-based batch (compaction force 40 kN; magnification: 53,490×); (**e**) the dextrates based-batch (compaction force 40 kN; magnification: 50,250×).The analysis confirmed that pores were of the sizes expected based on the mercury porosity measurement.

Figure 3 shows that a high porosity is associated with quicker disintegration times. The mannitol-based batches show the highest total porosities (13.13 ± 0.68% and 14.40 ± 0.12%, respectively) and exhibited the lowest median pore size (Table 1). The pore sizes were confirmed with scanning electron microscope pictures (Figure 4a–e).

### 2.3. Tablet Hardness

**Figure 5:**
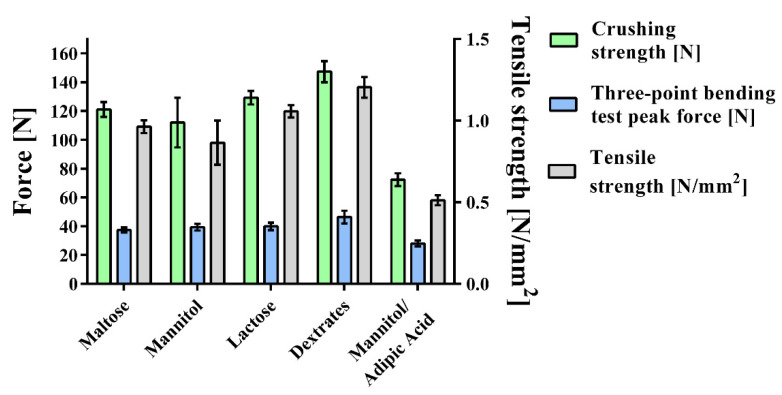
Tablet hardness: crushing strength was measured, and the three-point bending test was performed for each formulation (*n* =10). Tensile strength was calculated from the results.

To evaluate the hardness of the tablets, the three-point bending test was performed, and the friability of uncoated tablets and their resistance to crushing were measured (Figure 5, Table 3). The mannitol/adipic acid tablets showed the lowest values in all tests (crushing strength = 72.3 ± 4.4 N; tensile strength = 0.513 ± 0.031 N/mm^2^; three-point bending test peak force = 28.0 ± 2.1 N), which is not surprising, considering that the compaction force had to be decreased to 25 kN in this batch to avoid capping. All other batches were compacted with a compaction force of 40 kN. The dextrates-based tablets showed significantly higher values than the other batches (crushing strength = 147.4 ± 7.3 N; tensile strength = 1.204 ± 0.063 N/mm^2^; three-point bending test peak force = 46.3 ± 4.5 N). Moreover, it was the only batch that passed the test of friability showing 0.72% mass loss. None of the other tablets passed the friability test, which is to be expected for effervescent tablets due to their weights and tensile strengths.

#### 2.3.1. Three-Point Bending Test

The dextrates-based batches show the highest peak force of 46.3 ± 4.5 N (Figure 5, Table 3), which is significantly higher than the peak forces of the three other batches that were compacted with a compaction force of 40 kN. The peak forces for these batches were the following: 39.8 ± 2.6 N (lactose-based), 39.3 ± 2.3 N (mannitol-based), 37.4 ± 1.7 N (maltose-based). Within these batches, there were no significant differences in the measured peak forces. The lowest peak force was measured for the mannitol/adipic acid-based batch that was compacted with a force of 25 kN (28.0 ± 2.1 N).

#### 2.3.2. Friability of Uncoated Tablets

The dextrates-based batch showed a friability value of 0.72% (Table 3). For the other three batches, no values could be calculated, since tablets broke during the friability test. During the test of the lactose-based batch one tablet broke, three tablets from the mannitol-based batch broke, and during the test of the maltose-based batch five tablets broke. Since the mannitol/adipic acid tablets showed the lowest values during the other hardness tests, and since broken tablets could therefore be expected for this batch as well, the friability test was omitted for this batch.

#### 2.3.3. Resistance to Crushing

As in the other hardness tests, the dextrates-based batch showed the highest values for this test as well, breaking at significantly higher applied force (147.4 ± 7.3 N) than the other batches (Figure 5, Table 3). Once more, the lactose-based batch showed the second highest values (129.3 ± 4.7 N). However, this is not significantly higher than the values of the maltose-based batch (121.1 ± 5.1 N). The mannitol-based batch showed the lowest values (112.0 ± 17.3 N) of the batches that were compacted with 40 kN, significantly lower than the value for the lactose-based batches. Comparing the mannitol-based batch to the maltose-based batch, there was no significant difference. For the batch based on mannitol/adipic acid, a significantly lower breaking force was measured (72.3 ± 4.4 N).

#### 2.3.4. Tensile Strength

The tensile strength results for the different tablets mirrored those of resistance to crushing (Figure 5, Table 3). Dextrates-based tablets showed the highest values for tensile strength (1.204 ± 0.063 N/mm^2^), which were significantly higher than the values for lactose-based tablets (1.057 ± 0.038 N/mm^2^). There was also a significant difference between lactose- and maltose-based tablets (0.963 ± 0.039 N/mm^2^). Mannitol-based batches were characterized by significantly lower tensile strengths than the previously mentioned ones (mannitol (0.865 ± 0.135 N/mm^2^) and mannitol/adipic acid (0.513 ± 0.031 N/mm^2^)).

### 2.4. Stability

#### 2.4.1. Dynamic Vapor Sorption (DVS)

**Figure 6:**
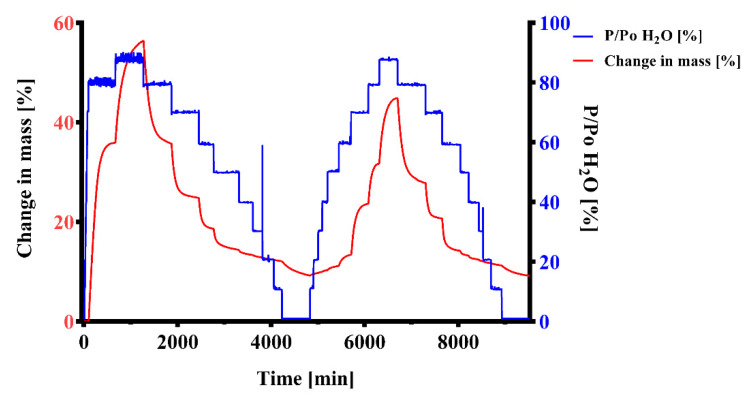
Dynamic vapor sorption change in mass analysis. An effervescent granule formulation containing mannitol as filler was investigated. Two cycles of absorption and desorption were performed, 0%-90%-0% P/P_0_ in 10% stages. The change in mass was recorded over time. A mass change dm/dt = 0.002% min^−1^ or 600 min (whichever occurred first) were selected as criteria for changing the humidity stage.

**Figure 7:**
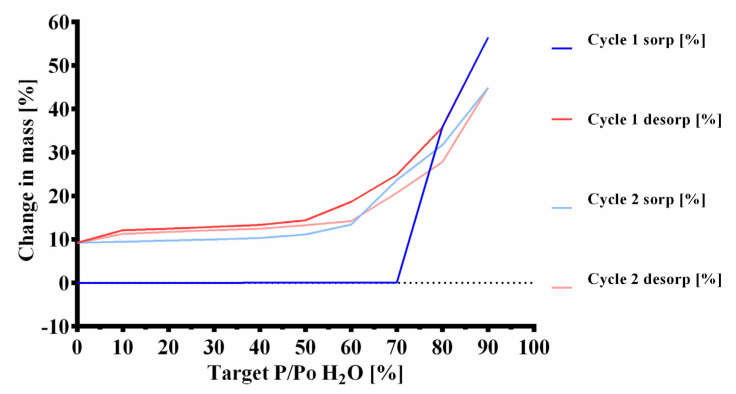
Dynamic vapor sorption isotherm analysis. Two sorption and desorption cycles (sorp = sorption; desorp = desorption) of an effervescent granule formulation containing mannitol as a filler from the same DVS measurement as in Figure 6 are displayed.

Effervescent formulations are usually very moisture-labile and require careful control of all manufacturing processes and conditions [45]. The formulations’ affinity and lability to humidity during processing and storage was evaluated with dynamic vapor sorption measurements. The sample of the mannitol-based effervescent granule mixture was found to have a high affinity for water. The initial mass of the sample (m_0_) was 16.0413 mg. The highest value in the first cycle (m_max1_) was 25.1013 mg, which was a mass increase of 56.5% compared to m_0_ (Figure 6 and Figure 7). After the first desorption cycle, the lowest value was 17.5162 mg (m_end1_) which is 109.2% of m_0_. In the second cycle, the weight increased to 23.2405 mg (m_max2_) which was a mass increase of 44.9% over m_0_ and an increase of 32.7% relative to the mass after the first cycle m_end1_. The mass at the end of the second cycle (m_end2_) was 17.5107 mg, 109.2% of m_0_.

#### 2.4.2. Bulk Stability Testing

**Figure 8:**
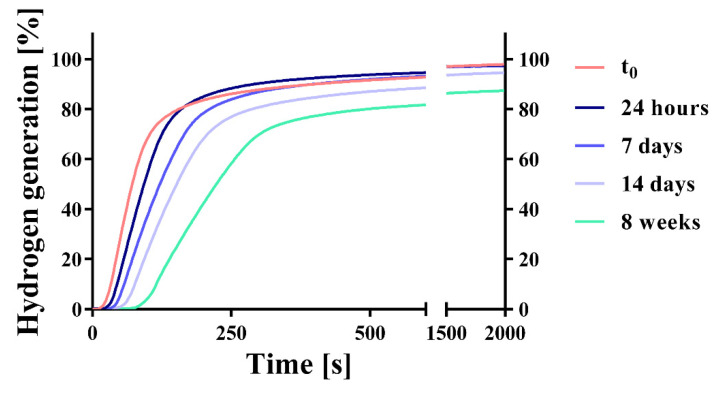
Kinetic hydrogen generation measurement (means; *n* = 3). Kinetics of hydrogen generation of unpacked tablets of the mannitol/adipic acid-based batch were measured at the starting point t_0_, and after 24 h, 7 days, 14 days, and 8 weeks of storage in a constant climate chamber (25 °C and 60% RH).

The total amount of generated hydrogen (Figure 8 and Table 4) was not significantly influenced by storage time from starting point t_0_ up to 14 days, and the differences were not significant. However, a slightly decreasing trend in hydrogen generation is visible. After 8 weeks, the amount of generated hydrogen decreased significantly. The tablets remained stable under the tested conditions for at least 14 days, but hydrogen generation was slower after longer storage times. f2 comparisons (Table 5) of tablets which were not stored (t_0_) or stored for 24 h can be considered similar. With increasing storage time, the hydrogen generation rate decreased, and the samples cannot be considered similar regarding f2 comparison. Tablet weight increased significantly at each sampling time.

### 2.5. Granular Flow Properties

Based on their angle of repose values, the granules showed good (maltose- and dextrates-based granules) to fair flow (lactose- and mannitol-based) [46]. The mannitol/adipic acid-based granule was not measured as it showed insufficient powder flow for the measurement. Flow through orifice values ranging from 7.9 s/100 g (maltose-based granule) to 11.5 s/100 g (lactose-based granules) were recorded. For mannitol-based batches, the powder flow had to be induced. The granules showed good (maltose-, dextrates-, and lactose-based granules) to fair (both mannitol-based granules) Hausner ratios and compressibility indices [46]. The granular characteristics are presented in Table 6.

## 3. Discussion

### 3.1. Content, Kinetic Hydrogen Generation, and Disintegration

Mannitol-based formulations generated the highest amount of hydrogen and showed the quickest disintegration and hydrogen generation (Figure 1, Figure 2 and Figure 3). Since mannitol is clearly not the most soluble of the investigated binders [47,48,49], the major factor responsible for this phenomenon was the porosity of the tablets (Figure 3; Table 1). Mannitol-containing tablets showed the highest porosity of all formulations, although this result can be partly attributed to the lower compaction force that was used for the mannitol/adipic acid-based batch in order to avoid capping. A higher porosity is commonly associated with a quicker disintegration time [50]. The median-sized pores measured by mercury porosimetry were confirmed with scanning electron microscopy (Figure 4a–e).

The rapid disintegration of the mannitol-based batches (Figure 1; Table 1) can also be attributed to the inter- and intragranular binding forces, which are the weakest among all fillers, as indicated by the lower hardness values of the tablets and the significantly higher amounts of fine particles (particle size d10: 14.0 ± 2.6 µm for mannitol/adipic acid-based tablets; 28.1 ± 3.5 µm for the mannitol-based batch, shown in Table 6). It is to be expected that the mannitol-based batches show the highest total porosities, while their median pore radius is significantly lower. It was previously shown that spray-dried mannitol processed by dry granulation yields tablets with favorable disintegration and acceptable hardness values, which is supported by our results [51]. The low SD of the batches’ hydrogen generation and magnesium content (Table 1) shows that dry granulation is suitable for avoiding the segregation of excipients during mixing that was observed during previous experiments using direct compaction. Moreover, no recycling and sieving of fines of the granules were performed, because this can have a negative impact on content uniformity [32]. The large differences between partial disintegration into granular particles and the complete disintegration time (Figure 1) suggest that strong deformation and bonding occurred during the dry granulation process of the maltose-, lactose-, and dextrates-based formulations resulting in high intragranular binding forces. The extra granular bonds that were formed during the tableting disintegrated first, most likely on the contact surfaces of the respective granules and Citrocoat^®^ N.

The different values for the hydrogen generation and the magnesium content determined via complexometric titration (Table 1) show that only mannitol-based batches reacted completely. The differences were probably caused by an incomplete reaction of magnesium, which could be related to the following observation: after the disintegration of the tablets manufactured with sodium stearyl fumarate, grey foam forms on top of the water, which resembles the color of the metallic magnesium particles that are most likely covered with sodium stearyl fumarate and are not able to react chemically. As a consequence of the medium’s slightly acidic pH-value, the stearyl fumarate anion is at least partly protonated, which decreases the agent’s solubility. The mannitol-based batches either do not contain sodium stearyl fumarate (mannitol/adipic acid-based batch) or else the disintegration takes place very quickly (mannitol) and the effect is not manifested. In this case, the time window available for the protonated stearyl fumarate to cover the surface of the magnesium particles and consequently to partly prevent the effervescent reaction before they have dissolved, is smaller.

### 3.2. Tablet Hardness

During formulation development it became clear that manufacturing tablets with good hardness values would be a challenging task (Table 3). Roller compaction causes a reduction in the tabletability of the granules compared to direct compression. However, roller compaction was necessary in order to avoid segregation of the powder particles. The phenomenon of loss of tabletability during roller compaction is known as work hardening and is considered to stem from increased resistance to deformation of a material during multiple compaction cycles [52,53,54,55]. Some authors view particle size enlargement and decreased surface area available for bonding during compaction as the main reasons for the loss of tabletability [56] while others [57,58] suggest that both mechanisms play a role. However, it is crucial to maintain a large enough particle size of the granules in order to avoid segregation after blending with Citrocoat^®^ N, which consists mainly of large particles (d50 = 405.3 µm) of high density. Moreover, a decreased specific surface area of the large particles is beneficial for the lubrication, since it can be expected that the effectiveness of the lubricant depends on the size of the particular surface area that is necessary to be covered [59]. Wet granulation was not considered as a good approach, because a high residual water content in the granules could exacerbate sticking of the formulation during tableting, as well as trigger the effervescent reaction.

In order to improve the hardness parameters (Figure 5; Table 3), an increased amount of diluent could be beneficial, since these substances show beneficial compactability characteristics. With reference to Heckel’s equation [60] to describe compaction mechanisms, the value of yield pressure was introduced [61] and values for yield pressure were characterized. Low values suggest soft and plastically deforming materials, while high values suggest brittle, fragmenting behavior and hard materials [62,63,64]. The yield pressure values depend on the experimental conditions so the values that are mentioned in different studies serve only as a rough guide [65]. Maltose is known to provide very good flow properties and shows good compactability [66]. Mannitol also shows good compactability [67], is moderately hard as a material, and shows a brittle compaction behavior, which is reflected by yield pressure values ranging from 132–135 MPa [63]. This is also reflected by its relatively lower lubricant sensitivity, which is a typical property of fragmenting materials, since they create new unlubricated bonding sites during fragmentation [68]. Lactose is a moderately hard to hard material according to its yield pressure of 174–233 MPa [69], which is known to consolidate with an initial fragmentation step and deform plastically on newly created bonding sites and within amorphous regions [70,71]. Dextrates shows a more plastic deformation (yield pressures: 67–166 MPa) as well as some fragmentation during compaction, properties which depend on the formulation before compaction, and the material can be compacted into tablets of high strength [72,73,74,75]. According to its yield pressure it can be classified as moderately hard. Hence, it can be concluded that a larger amount of the investigated saccharide fillers would increase the hardness of the formulations although their deformation mechanisms differ slightly. Specifically, the addition of spray-dried maltose to a granular mannitol blend has proven to increase tablet hardness; furthermore, owing to its spherical morphology and good compactability, decreased capping and enhanced flow properties have also been attributed to this filler [66]. However, most studies have investigated directly compactable or wet granulated materials. Based on the particle sizes of the roller-compacted granules, it can be concluded that the mannitol-based batches showed a lower compactability and/or a higher granular fragmentation during the milling process since they exhibited significantly lower d10 particle size values. The lower hardness values and the high number of fine particles correspond excellently with the fact that mannitol-based batches show the highest total porosity while also showing the lowest average pore size.

To improve tablet hardness, especially of the mannitol/adipic acid-based batch, the citric acid excipient Citrocoat^®^ N (granule with citric acid core coated with a monosodium citrate shell) could be swapped for a compound excipient like citric acid DC, directly compactable citric acid with maltodextrin coating. However, sticking during compaction, which was resolved by the use of Citrocoat^®^ N, would reoccur. Another possibility would be to combine the investigated saccharide fillers in a DOE experiment to find an optimal compromise between improved hardness, good disintegration, and hydrogen generation. The inclusion of an extra granular dry binder might also be useful in this regard. The hardness tests of the different batches showed clearly that the dextrates-based batch was the most favorable filler with respect to hardness parameters.

### 3.3. Dynamic Vapor Sorption (DVS)

In the first stages of humidity (0–70% P/P_0_ H_2_0 at 25 °C) no mass changes greater than the near-equilibrium state criterium (dm/dt = 0.002% min^−1^) were observed (Figure 6 and Figure 7). In previous runs, the sorption started already at 70% P/P_0_ H_2_0. In the later stages, starting from 80% P/P_0_ H_2_0 a large increase of mass was observed. Up to 80% P/P_0_ H_2_0 at 25 °C only physisorption occurs, with bulk sorption becoming subsequently dominant [76,77]. This indicates that the hygroscopic Citrocoat^®^ N had a higher resistance towards humidity than regular anhydrous citric acid, which is known to start sorption of water at 62% P/P_0_ H_2_0 at 25 °C [78]. However, the large maximal mass (m_max1_ = 156.5% of m_0_) and the mass at the end of the measurement (m_end2_ = 109.2% of m_0_) show that the powder has a high affinity for humidity. The gap between the peak values for m_max_ (m_max1_ = 156.5% and m_max2_ = 144.9%) of the two cycles is too large to be explained by gas generation on account of the low percentage by mass of magnesium in the tablet. The most probable explanation lies in the possible partial dissolution and recrystallization at interparticulate interfaces, which would result in a reduced total surface area available for water sorption. If there was no chemical reaction, two similar sorption and desorption cycles would be expected. No chemical change takes place after cycle 1, since a regular sorption and desorption cycle with m_end1_ ≈ m_end2_ can be observed. Comparison of the sorption cycles reveals that, during the second cycle of sorption, the mass of the powder increased at earlier humidity stages (4.17% mass increase at 60% P/P_0_ H_2_0) than in the first cycle (0.04% mass increase at 60% P/P_0_ H_2_0). This indicates that the protective monosodium citrate coating from Citrocoat^®^ N has been disrupted most probably either through partial dissolution in the adsorbed water film, or through a change in the crystal form, or a combination of both.

### 3.4. Bulk Stability Testing

Data from the dynamic vapor sorption experiments suggested that the investigated mannitol/adipic acid-based formulation was stable up to 25 °C; 60% RH for the selected stability criteria dm/dt = 0.002% min^−1^ (Figure 6 and Figure 7). During the bulk stability experiment (Figure 8, Table 4) the tablets were exposed to these conditions for up to 8 weeks. During this time, the tablets continuously increased in weight through water sorption. However, the amounts of water taken up were very low and did not cause an extensive reaction in the tablet, at least not in the first 14 days. Upon exposure to humidity, magnesium reacts to form magnesium hydroxide (Mg(OH)_2_), which passivates its surface [37,38,39,40,41,42,43]. Mg(OH)_2_ is not water-soluble so the surface has to be reactivated. Low pH values as well as organic ligands like citrate or ascorbate are known to enhance the dissolution of Mg(OH)_2_ and thereby restore the active magnesium surface. Since the amount of generated hydrogen, as well as the rate of generation, decreased during storage time it can be expected that those magnesium surfaces exposed to the gaseous environment (particularly those near the tablet surface) were markedly passivated. This explains the slight reduction in hydrogen generated as well as the decreased hydrogen generation rate, since the passivated surfaces have to be reactivated for the effervescent reaction to start. Furthermore, potential partial solution and recrystallization of Citrocoat^®^ N could lead to a decrease in the specific surface area of the tablet available for effervescent reaction. The significantly lower hydrogen generation after 8 weeks demonstrates that the reaction continues under these conditions (25 °C; 60% RH), although it is very slow. During the whole duration of exposure to these conditions, the tablets continued to gain weight by water vapor sorption, which enabled the continuation of the reaction. In contrast to conventional effervescent tablets, where additional water is a byproduct of their effervescent reaction [29], the effervescent hydrogen-generating tablets do not generate additional water. Without this autocatalytic reaction enhancement, the effervescent hydrogen-generating tablets could be regarded as slightly less moisture-labile than similarly manufactured conventional effervescent tablets. The experiment clearly showed that the tablets need adequate packaging and controlled environmental conditions for processing and packaging of the tablets and the intermediate products. For primary packaging, we recommend plastic tubes with drying agents in the cap or aluminum/aluminum (alu/alu) blister packs in case that a single unit packaging is preferred. Both options would offer sufficient moisture protection.

### 3.5. Granular Flow Properties

Granule flow was sufficient for subsequent tablet manufacturing (Table 6). The tablets are characterized by low SD in magnesium content and weight deviation (Table 1 and Table 3). The mannitol-based batches exhibited an inferior powder flow, which is probably related to their high number of fine particles (see d10 particle size values). The batch lubricated with adipic acid (d50 value: < 15 µm) was particularly affected since its level of lubricant is much higher (10% adipic acid addition compared to 0.5% for the other batches). However, the good (maltose-, dextrates-, and lactose-based granules) to fair (both mannitol-based granules) Hausner ratios and compressibility indices as well as the high bulk density ensured a constant filling process of the die of the tablet press.

## 4. Materials and Methods

### 4.1. Materials

Magnesium powder (−325 mesh, 99.8% purity) was purchased from Alfa Aesar (Heysham, England). Citrocoat^®^ N (granule with citric acid core coated with a monosodium citrate shell) was kindly gifted by Jungbunzlauer Suisse AG (Basel, Switzerland). L(+)-ascorbic acid was purchased from Carl Roth GmbH + Co. KG (Karlsruhe, Germany). Samples of Advantose^®^ 100 (maltose) and Kerry Lactose Anhydrous NF DT High Velocity (lactose) were received as a gift from Lehmann&Voss&Co. KG (Hamburg, Germany). Mannogem EZ^®^ (mannitol) was kindly donated by Spi Pharma (Wilmington, NC, USA). Adipic acid Emprove^®^ Essential was generously donated by Merck KGaA (Darmstadt, Germany). Emdex^®^ (dextrates, glucose monohydrate and different polysaccharides derived from starch according to USP 42-NF 37) and Pruv^®^ (sodium stearyl fumarate) were received as a gift from JRS Pharma GmbH + Co. KG (Rosenberg, Germany). Stochiometric quantities of each excipient included in one tablet of the respective batches are listed in Table 7.

### 4.2. Sieving of Powders

The saccharide fillers (maltose, mannitol, lactose, and dextrates) and ascorbic acid were hand-sieved (400 µm or 800 µm sieve mesh size) to disaggregate any agglomerates.

### 4.3. Milling of Adipic Acid

Adipic acid (Emprove^®^ Essential) was milled with a Fritsch Pulverisette Type 00.001 (Fritsch GmbH, Idar-Oberstein, Germany) single ball mill (70 mm agate grinding ball in 95 mm agate grinding bowl; intensity level 10; 3 h). 15 g of adipic acid were milled per cycle and united in a closed vessel afterwards

### 4.4. Blending of Powders

The powder formulations (50 g of magnesium powder, 12 g of ascorbic acid, and 457.65 g of the respective saccharide fillers (maltose, mannitol, lactose, and dextrates)) were blended with a Turbula^®^ 3D shaker mixer T 2 F (Willy A. Bachofen GmbH, Muttenz, Switzerland), equipped with a 1.6 L mixing basket for 10 min at 49 rpm.

### 4.5. Roller Compaction/Dry Granulation

Dry granulation of the blended powder from Section 4.4. was conducted on a roller compactor (TFC-LAB Micro) by Freund-Vector Corp. (Marion, OH, USA), which was equipped with standard compacting rolls “S” (diameter of 50 mm; width 24 mm) by Freund-Vector Corp. (Marion, OH, USA). As input parameters, a compaction force of 4 kN which equals $1.\bar{6\;}$ kN/cm for the used compacting roll, roll speed of 1 rpm, and a screw speed of 30 rpm, were used to compact the powder to ribbons.

### 4.6. Dry Cone Milling

Milling of the ribbons obtained from 4.5. was performed immediately after previous roller compaction step with a U5 Quadro Comil from Quadro Engineering Corp. (Waterloo, ON, Canada) with a 1575 µm rasp mesh screen at 1000 rpm.

### 4.7. Blending of Dry Granules with Citrocoat^®^ N

After dry roller compaction and cone milling, 207.86 g of the dry granules and 192.14 g of Citrocoat^®^ N were blended in a Turbula^®^ 3D shaker mixer T 2 F for 10 min.

### 4.8. Addition of Lubricant

2 g of sodium stearyl fumarate (Pruv^®^) or 40 g adipic acid (Emprove^®^ Essential) were sieved onto 400 g of the blend of dry granules with Citrocoat^®^ N using a 100 µm or 160 µm mesh sieve to disaggregate any agglomerates. Consequently, either 0.5% or 10% of lubricant were added to the granules. Afterwards, the mixture was blended once more with a Turbula^®^ 3D shaker mixer T 2 F for 3 min.

### 4.9. Compaction of Tablets

**Figure 9:**
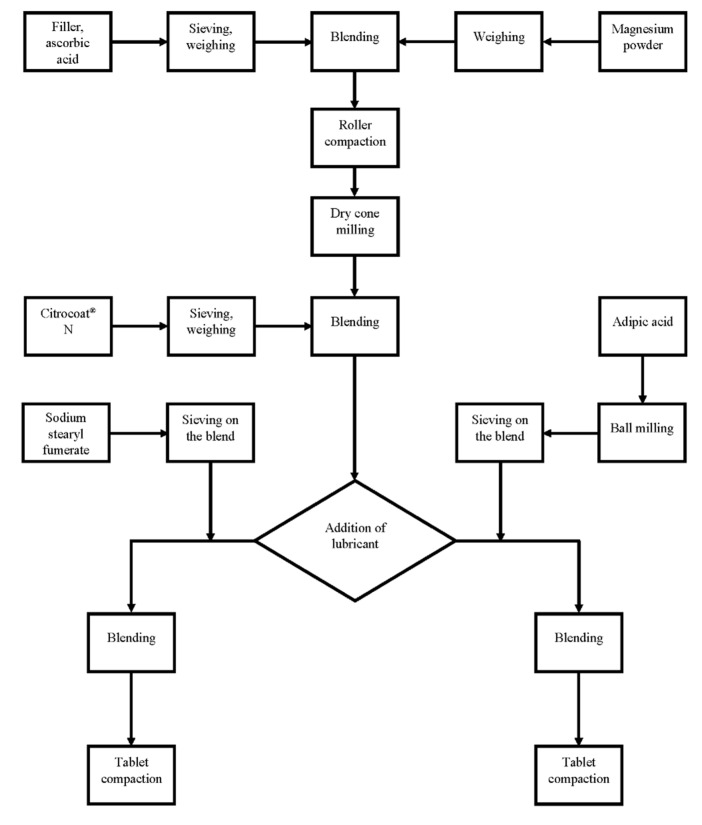
Flow diagram of the manufacturing process.

Tablets were compacted on an instrumented Korsch EK0 eccentric tablet press (Korsch AG, Berlin, Germany) with a Korsch steel punch set (diameter 18 mm). Tablets were compacted at 25 kN (mannitol/adipic acid) or 40 kN (maltose, mannitol, lactose, dextrates). The compaction forces were recorded and processed with a Spider 8 electronic measuring system and Catman 4.5 software (Hottinger Baldwin Messtechnik GmbH, Darmstadt, Germany). 402 g of the granules lubricated with sodium stearyl fumarate (Pruv^®^) or 440 g of the granules lubricated with adipic acid (Emprove^®^ Essential) were used for the compaction process. Since every tablet should contain 75 mg of magnesium which is included in 1.5g of unlubricated granules, the theoretical tablet weights were 1.5075 g (granules + 0.5% lubricant) for the granules lubricated with sodium stearyl fumarate (Pruv^®^) and 1.65 g (granule + 10% lubricant) for the granules lubricated with adipic acid (Emprove^®^ Essential). The measured tablet weights are presented in Table 3. Figure 9 shows a flow diagram that summarizes all manufacturing processes. 

### 4.10. Kinetic Hydrogen Generation Measurement

Kinetic hydrogen generation measurement was conducted with slight modifications to the approach of Brack et al. [79]. Specifically, measurement of hydrogen evolution was similarly performed using a volumetric apparatus and a data logging scale, while the chemical reaction differed to the one in the above-cited article. Brack et al. measured hydrogen that is generated by the reaction of silicon with aqueous sodium hydroxide solutions; whereas, in the present study, hydrogen generated by the reaction of magnesium with the organic acids in the formulations was determined.

A 50 mL Erlenmeyer flask filled with 30 mL of deionized water was used for the chemical reaction. A plastic tube with an inner diameter of 3 mm and a length of 53 cm was used to direct the evolving hydrogen gas to a 250 mL measuring cylinder which was filled with 170 mL of water. The water bath was filled until it flooded slightly. Then, 30 mL of water was added, which was followed by a refractory period of 30 min to make sure that the water bath was completely leveled. Experiments were conducted at room temperature (23 ± 1 °C). The tablets were put into the Erlenmeyer flask, the beaker was closed immediately, and the water displacement was recorded with a data logging Denver S 2002 scale (Denver Instruments, Bohemia, NY, USA) using Sartorius Wedge recording software (Sartorius AG, Goettingen, Germany). When 90% of the expected amount of water was displaced or after 400 s (whichever occurred first), the solution was stirred to recover as much hydrogen as possible.

The volume of displaced water is equivalent to the volume of hydrogen. The amount of hydrogen can then be calculated from its volume employing the equations:

(2) $$p*V=m_{Hydrogen}*R_{s}*T\;$$

p is pressure [kPa]; V is volume $\left[m^{3}\right]$; $m_{hydrogen}$ is mass of hydrogen [kg]; $R_{s}\;$ is specific gas constant of hydrogen [J/kgK]; T is temperature [K].

(3) $$V\;=\frac{m_{water}}{\rho_{water}}$$

V is volume $\left[m^{3}\right]$; $m_{hydrogen}$ is mass of hydrogen [kg]; ρ is density of water (25 °C $\left[997\;\mathrm{kg}/m^{3}\right]$).

(4) $$p*\frac{m_{water}}{\rho_{water}}=m_{Hydrogen}*R_{s}*T$$

(5) $$m_{Hydrogen}=\frac{p\frac{m_{water}}{\rho_{water}}}{R_{s}T}$$

f2 values of the hydrogen generation profiles were calculated as suggested by Moore and Flanner [80] in order to evaluate the similarity of the hydrogen generation profiles of the different formulations.

### 4.11. Magnesium Content (Complexometric Titration)

The magnesium content of the tablets was determined according to Ph. Eur. 10.1; 2.5.11. (complexometric titrations/magnesium). A buffered sample was titrated with sodium edetate (0.1 mol/L). Mordant black was used as a color indicator. For enhanced visibility, 75 mg mordant black was used. A correction factor of 0.998 was determined (*n* = 5) by titrating 75 mg of magnesium powder. *n* = 5 was also used for each batch of tablets.

### 4.12. Disintegration

Disintegration experiments were performed in a beaker with 200 mL of water as described in the monograph of effervescent tablets Ph. Eur. 10.1/0478. Primary and secondary endpoints of disintegration were determined visually. The first measurement was taken when the tablet had disintegrated partially into granular particles. The second measurement was taken as the endpoint, after the tablet and the particles had disintegrated completely.

### 4.13. Three-Point Bending Test

The three-point bending test was performed on a Texture Analyzer TA.XTplus equipped with a Three Point Bend Rig ((HDP/3PB) both from Stable Micro Systems Ltd. (Surrey, UK)). Ten measurements of the peak force were performed for each batch. The loading pin was programmed to move 0.05 mm/s starting at a trigger force of 1 N. The gap between the supporting pins was 14 mm.

### 4.14. Friability of Uncoated Tablets

The friability of the dedusted tablets was measured with a TAP friability tester by Erweka (Langen, Germany). One hundred rotations were performed at 21 rpm. Afterwards, the tablets were dedusted again. Tablets were weighed prior to and after the test, and the loss of mass (friability) in % was determined.

### 4.15. Resistance to Crushing

Resistance to crushing was measured as described in Ph. Eur. 10.1/2.9.8 with a PTB-M-manual tablet hardness testing instrument by Pharma Test Apparatebau AG (Hainburg, Germany). Ten measurements were performed on every batch. Prior to this test, every tablet was weighed.

### 4.16. Tensile Strength

Tensile strength (*σ*) was calculated as suggested by Fell et al. [81]:

(6) $$\sigma =\frac{2P}{\pi DT}\;$$

P  is crushing strength [N]; D is tablet diameter [mm]; T is tablet thickness [mm].

Crushing strength was measured with the PTB-M-manual tablet hardness testing instrument from Pharma Test Apparatebau AG (Hainburg, Germany) as mentioned above, and tablet thickness was measured with a ID-C112XBS thickness gage (Mitutuyo Corporation, Kanagawa, Japan).

### 4.17. Porosity and Pore-Size Distribution of Solids by Mercury Porosity

Porosity measurements were performed with Pascal 140 and Pascal 240 porosimeters from Thermo Fisher Scientific (Waltham, MA, USA). The tablets were cut with a band saw in order to fit them into the porosimeter. In the Pascal 140 device, the sample is exposed to mercury at increasing pressures up to 400 kPa. Afterwards, the sample is transferred to the Pascal 240 device and the pressure is increased to 200 MPa.

### 4.18. Helium Pycnometry

True density was measured using a helium pycnometer AccuPyk II 1340 V2.1 (Micrometrics Instrument Corporation; Norcross, GA, USA). Three tablets per batch were analyzed (134.4 kPa helium gas pressure for analysis; 15 purges; equilibration rate: 0.0345 kPa/min).

### 4.19. Particle Size Analysis by Laser Light Diffraction

Particle sizes were measured according to Ph. Eur. 10.1/2.9.31.with a LS 13 320 laser diffraction particle sizing analyzer equipped with a Tornado DPS module (Beckman Coulter, Inc.; Brea, CA, USA). The Fraunhofer method was used to calculate the particle sizes.

### 4.20. Bulk Density and Tapped Density of Powders

Bulk and tapped densities were measured according to Ph. Eur. 10.1/2.9.34; method 1. Therefore, 150 g of sample were used as suggested for samples with high density. Tapped density was measured with a Engelsmann jolting volumeter type EU42E2/114S-WF from J. Engelsmann AG (Ludwigshafen, Germany). The compressibility index and Hausner ratio were calculated as suggested. Each measurement was performed three times.

### 4.21. Angle of Repose

Angle of repose was determined according to Ph. Eur. 10.1/2.9.36 using a funnel and the drained angle of repose method. The measurements were conducted three times with a PTG S3 powder analysis device (Pharma Test Apparatebau AG; Hainburg, Germany)

### 4.22. Flowability

Flowability was determined according to Ph. Eur. 10.1/2.9.16 using nozzle 1. Triplicate measurements were conducted with a PTG S3 powder analysis device (Pharma Test Apparatebau AG; Hainburg, Germany)

### 4.23. Loss on Drying Analysis of the Granules

Loss on drying analysis was conducted with a Precisa moisture analyzer XM60 (Precisa Gravimetrics AG; Dietikon; Switzerland). About 1 g of granule was weighed, then the device was heated to 105 °C. After 2 min, the weight loss (%) was measured. The measurements were performed three times.

### 4.24. Dynamic Vapor Sorption (DVS)

DVS measurements were performed with a DVS Advantage device (Surface Measurement Systems Ltd., London, UK). An effervescent granule formulation containing mannitol was investigated. Two cycles of sorption and desorption were performed, 0%-90%-0% P/P_0_ H_2_0 in 10% increments. Criteria for changing the increment were a mass change smaller than dm/dt = 0.002% min^−1^ [82], stable for 10 min. If the dm/dt criterion was not met, the increment was changed after 600 min.

### 4.25. Bulk Stability Testing

Tablets of the mannitol/adipic acid-based batch were investigated. Tablets were stored in a KBF P 240 constant climate chamber (Binder GmbH, Tuttlingen, Germany) at 25 °C and 60% RH for 0 h, 24 h, 7 days, 14 days, and 8 weeks, respectively. Afterwards, weight gain and kinetic hydrogen generation were measured. The kinetic hydrogen generation measurement was performed as described above. Weight gain was measured on an analytical scale (Sartorius LE225D-0CE; Sartorius AG, Goettingen, Germany).

### 4.26. Scanning Electron Microscopy

To investigate the tablet surface, the tablets were coated with a gold layer (20 nm thickness) using a sputter coater SCD 050 (Bal-Tec AG; Balzers, Liechtenstein) and stored in an oven for 24 h (70 °C). The samples were investigated with a Leo 1530 scanning electron microscope (Carl Zeiss Microscopy GmbH; Jena, Germany) using an electron high tension of 15 kV for the mannitol/adipic acid-based and 5 kV for all other batches.

### 4.27. Statistical Analysis

IBM SPSS statistics (IBM Corporation; Armonk, NY, USA) was used for statistical calculations of the obtained data. Results were compared with ANOVA. Significance was assumed for *p*-values < 0.05.

## 5. Conclusions

Through careful selection of excipients and process conditions, we could successfully formulate an effervescent hydrogen-generating tablet. Challenges associated with the manufacturing processes—such as segregation, sticking, and slow disintegration—could all be solved. Mannitol-based formulations showed the quickest disintegration and satisfactory hardness values, and these formulations were therefore selected for further development and stability testing. Dextrates-based formulations performed best regarding hardness and facilitated a precise and rapid generation of hydrogen. As expected, this formulation proved to be moisture-labile. For this reason, we recommend a strict monitoring of the processing and storage conditions as well as careful choice of the primary packaging materials.

## Figures and Tables

**Table 1:** Content, kinetic hydrogen generation, and disintegration displayed as mean and (SD).

Parameter	Maltose	Mannitol	Mannitol/ Adipic Acid	Lactose	Dextrates
H_2_ generation (mg)	5.887 (0.027)	6.488 (0.163)	6.589 (0.129)	5.719 (0.099)	5.794 (0.110)
H_2_ generation (%)	94.64 (0.43)	104.30 (2.63)	105.91 (2.08)	91.93 (1.59)	93.13 (1.76)
H_2_ generation (%) excluding weight incorrectness	92.28 (0.28)	102.80 (2.93)	103.89 (1.77)	92.23 (1.61)	92.61 (1.18)
Mg; complexometric titration (mg)	76.13 (0.36)	78.02 (1.92)	78.70 (1.13)	75.40 (1.28)	80.16 (1.46)
Mg; complexometric titration (%)	101.51 (0.48)	104.03 (2.56)	104.94 (1.51)	100.54 (1.70)	106.88 (1.95)
Partial disintegration into granular particles (mm:ss)	03:11(00:06)	01:05 (00:04)	01:08 (00:05)	03:04 (00:05)	03:11 (00:06)
Disintegration time (mm:ss)	03:58 (00:25)	01:12 (00:03)	01:23 (00:03)	05:00 (00:32)	04:17 (00:15)
Porosity by Hg intrusion (%)	10.33 (0.29)	13.13 (0.68)	14.40 (0.12)	12.52 (0.23)	11.4 (0.79)
Median pore radius (μm)	0.1912 (0.0124)	0.0502 (0.0073)	0.0745 (0.0294)	0.1330 (0.0143)	0.1545 (0.0151)

**Table 2:** f2 comparison. Kinetic hydrogen generation measurements of tablet batches manufactured with different ex-cipients were compared with the f2 comparison; according to SUPAC guidelines release profiles of tablets with a f2 value between 50 and 100 are considered similar.

Excipient	Maltose	Mannitol	Mannitol/ Adipic Acid	Lactose	Dextrates
Maltose		16.08	16.18	64.24	44.18
Mannitol	16.08		85.95	15.21	13.60
Mannitol/Adipic Acid	16.18	85.95		15.62	14.59
Lactose	64.24	15.21	15.62		54.65
Dextrates	44.18	13.60	14.59	54.65	

**Table 3:** Tablet hardness characteristics displayed as mean and (SD).

Parameter	Maltose	Mannitol	Mannitol/ Adipic Acid	Lactose	Dextrates
Crushing strength (N)	121.1 (5.1)	112.0 (17.3)	72.3 (4.4)	129.3 (4.7)	147.4 (7.3)
Tensile strength (N/mm^2^)	0.963 (0.039)	0.865 (0.135)	0.513 (0.031)	1.057 (0.038)	1.204 (0.063)
Three-point bending test peak force (N)	37.4 (1.7)	39.3 (2.3)	28.0 (2.1)	39.8 (2.6)	46.3 (4.5)
Friability (%)	broken tablets	broken tablets	not tested	broken tablets	0.72
True density (g/mL)	1.624 (0.13)	1.581 (0.007)	1.569 (0.003)	1.603 (0.025)	1.633 (0.006)
Weight (g)	1.545 (0.011)	1.537 (0.019)	1.631 (0.007)	1.495 (0.005)	1.515 (0.011)

**Table 4:** Hydrogen generation and weight gain of the bulk stability samples expressed as mean and (SD).

Storage Time	H_2_ Generation (%)	Weight Gain (%)
No storage	97.86 (2.98)	-
24 h	97.55 (3.04)	0.504 (0.032)
7 days	97.31 (1.24)	1.029 (0.005)
14 days	94.52 (1.84)	1.289 (0.092)
8 weeks	87.33 (1.47)	2.039 (0.161)

**Table 5:** f2 comparison. Kinetic hydrogen generation measurements of the bulk stability samples were compared with the f2 comparison; according to SUPAC guidelines release profiles of tablets with a f2 value between 50 and 100 are considered similar.

Storage Time	t_0_	24 h	7 Days	14 Days	8 Weeks
t_0_		51.41	36.07	26.77	17.00
24 h	51.41		45.65	31.54	19.49
7 days	36.07	45.65		48.63	26.76
14 days	26.77	31.54	48.63		38.95
8 weeks	17.00	19.49	26.76	38.95	

**Table 6:** Granular characteristics displayed as mean and (SD).

Parameter	Maltose	Mannitol	Mannitol/ Adipic Acid	Lactose	Dextrates
Angle of repose (°)	32.47 (0.3)	39.3 (0.8)	-	35.7 (0.6)	33.4 (1.0)
Flow through an orifice (s/100 g)	7.9 (0.1)	-	-	11.5 (0.5)	9.6 (0.0)
Bulk density (g/mL)	0.872 (0.000)	0.795 (0.005)	0.803 (0.005)	0.869 (0.012)	0.855 (0.006)
Tapped density (g/mL)	0.974 (0.000)	0.949 (0.000)	0.991 (0.008)	1.005 (0.008)	0.970 (0.007)
Hausner ratio	1.12 (0.00)	1.19 (0.01)	1.23 (0.01)	1.16 (0.03)	1.13 (0.01)
Compressibility index (%)	10.5 (0.0)	16.3 (0.5)	18.9 (0.5)	13.5 (1.6)	11.8 (0.6)
Particle size d10 (µm)	130.9 (11.9)	28.1 (3.5)	14.0 (2.6)	56.4 (3.0)	90.5 (12.6)
Particle size d50 (µm)	463 (22.6)	461.9 (18.4)	457.28 (15.2)	429.9 (12.3)	458.3 (23.7)
Particle size d90 (µm)	1411.4 (117.4)	1428.6 (40.3)	1430.6 (41.6)	1507.8 (40.0)	1500.2 (116.1)
Loss on drying (%)	1.80 (0.06)	1.03 (0.11)	1.02 (0.18)	1.29 (0.11)	4.32 (0.20)

**Table 7:** Stochiometric quantity of each excipient included in one tablet of the respective batches.

Excipient/Tablet (mg)	Maltose	Mannitol	Mannitol/ Adipic Acid	Lactose	Dextrates
Magnesium powder	75	75	75	75	75
Ascorbic acid	18	18	18	18	18
Citrocoat^®^ N	721	721	721	721	721
Maltose	686				
Mannitol		686	686		
Lactose				686	
Dextrates					686
Sodium stearyl fumarate	7.5	7.5		7.5	7.5
Adipic acid			150		

## Data Availability

Data is contained within the article.
